# Partial cystectomy for a primary locally advanced leiomyosarcoma of the bladder: a case report and review of the literature

**DOI:** 10.1002/ccr3.1476

**Published:** 2018-03-13

**Authors:** Umesh Jayarajah, Manoj Hilary Fernando, Kasun Bandara Herath, Vipula Chandu de Silva, Serozsha Anura Sahadeva Goonewardena

**Affiliations:** ^1^ Department of Urology National Hospital of Sri Lanka Colombo Sri Lanka; ^2^ Department of Pathology Faculty of Medicine University of Colombo Sri Lanka

**Keywords:** case report, partial cystectomy, primary leiomyosarcoma of the bladder

## Abstract

Partial cystectomy with wide local excision may be considered a suitable option for selective cases of locally advanced bladder leiomyosarcoma without evidence of distant metastasis; thereby preserving the functional outcome and quality of life. A negative margin, complete tumor resection, and frequent follow‐up in such patients are mandatory.

## Background

Nonepithelial tumors of the urinary bladder are rare and account for <5% of all bladder malignancies. Leiomyosarcoma is even sparse being 0.1% of overall bladder cancers 1. Due to the rarity of the disease, the knowledge related to the natural history and prognosis is poor. Furthermore, there is no consensus regarding the standard treatment protocol. The leiomyosarcomas of the bladder are usually aggressive tumors with very poor prognosis usually managed with radical cystectomy 1, 2. We report a case of a 54‐year‐old woman with locally advanced leiomyosarcoma of the bladder who was successfully treated with partial cystectomy and wide local excision.

## Case Presentation

**Figure 1:**
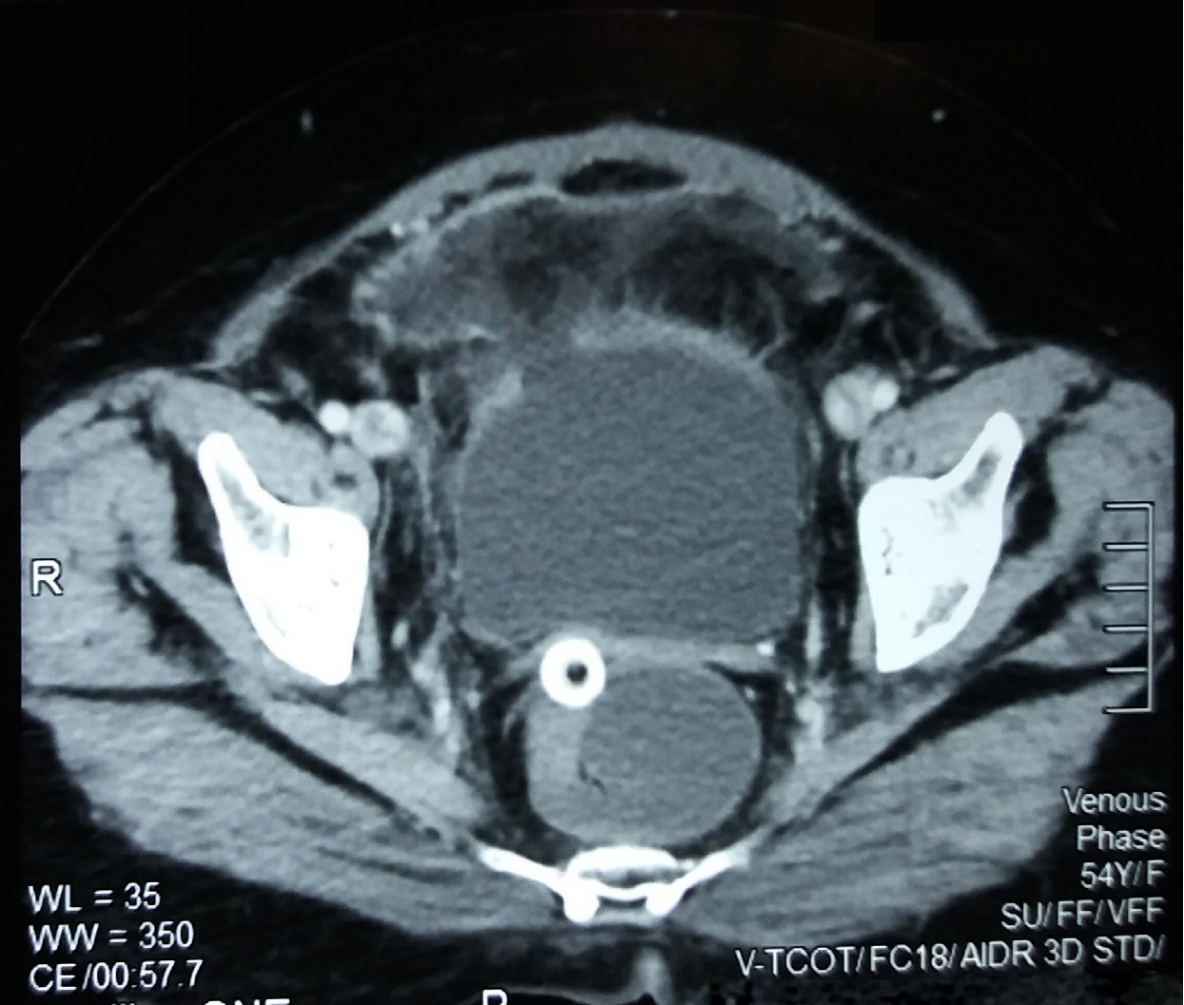
CT scan (Computed tomography) of the abdomen showing a 5.0 cm (Anteroposterior) × 7.0 cm (Transverse) × 7.0 cm (craniocaudal) space‐occupying lesion in the anterior wall of the bladder and the anterior abdominal wall with adjacent irregularly thickened bladder wall showing contrast enhancement.

A 54‐year‐old woman presented with a history of painful hematuria for 2 weeks associated with lower abdominal pain. There were no other associated lower urinary tract symptoms. The rest of the history was unremarkable. The basic biochemistry was within normal limits. A CT scan (computed tomography) of the abdomen was performed, which showed a 5.0 cm (anteroposterior) × 7.0 cm (transverse) × 7.0 cm (craniocaudal) space‐occupying lesion in the anterior wall of the bladder and the anterior abdominal wall. The mass had soft tissue and fat densities. Bladder wall adjacent to the mass was irregularly thickened and showed contrast enhancement. Both kidneys and upper tracts appeared normal, and no evidence lymph node involvement or distant metastasis was noted (Fig. 1).

**Figure 2:**
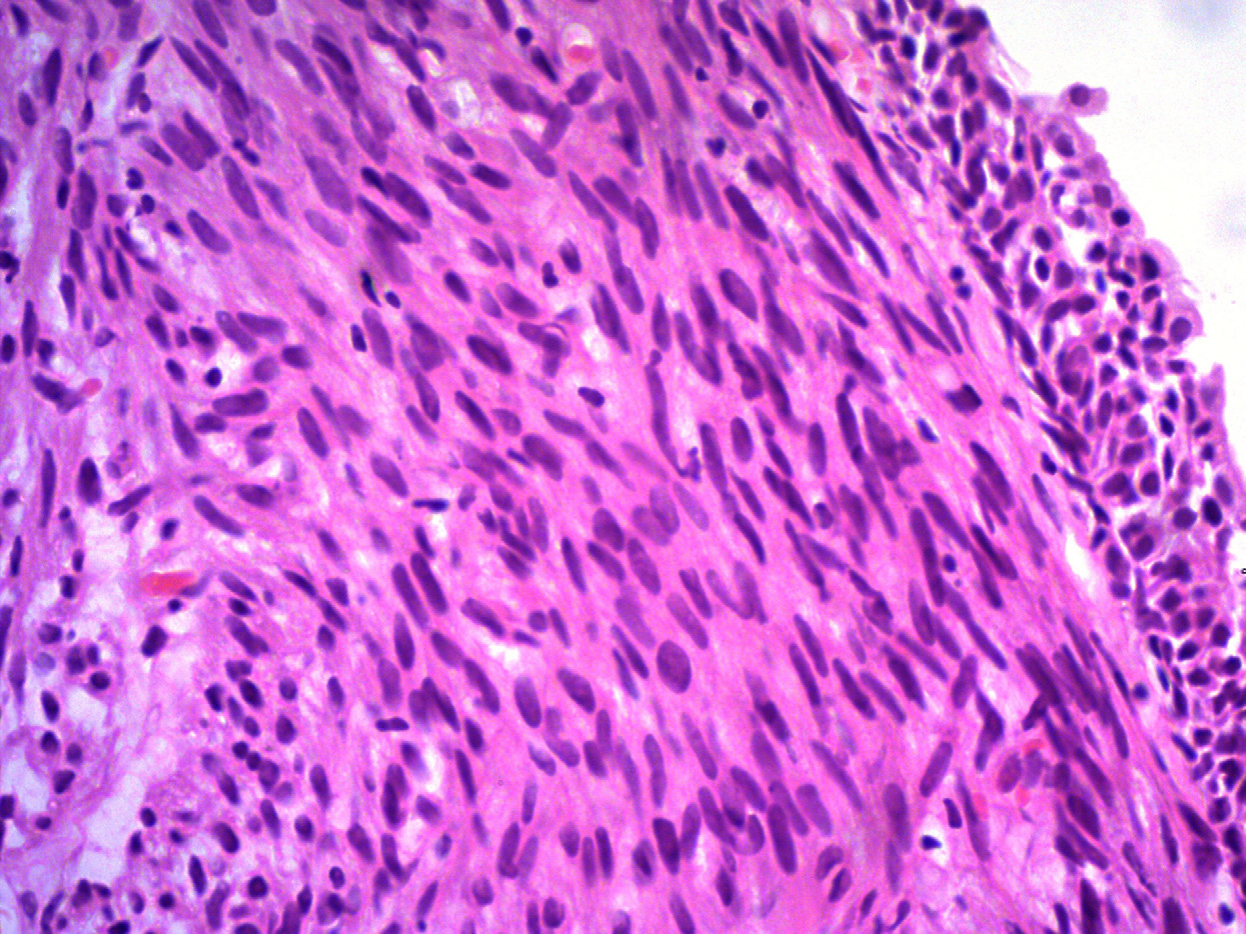
H and E staining viewed under × 40 showing a spindle cell tumor, lined by normal urothelium.

**Figure 3:**
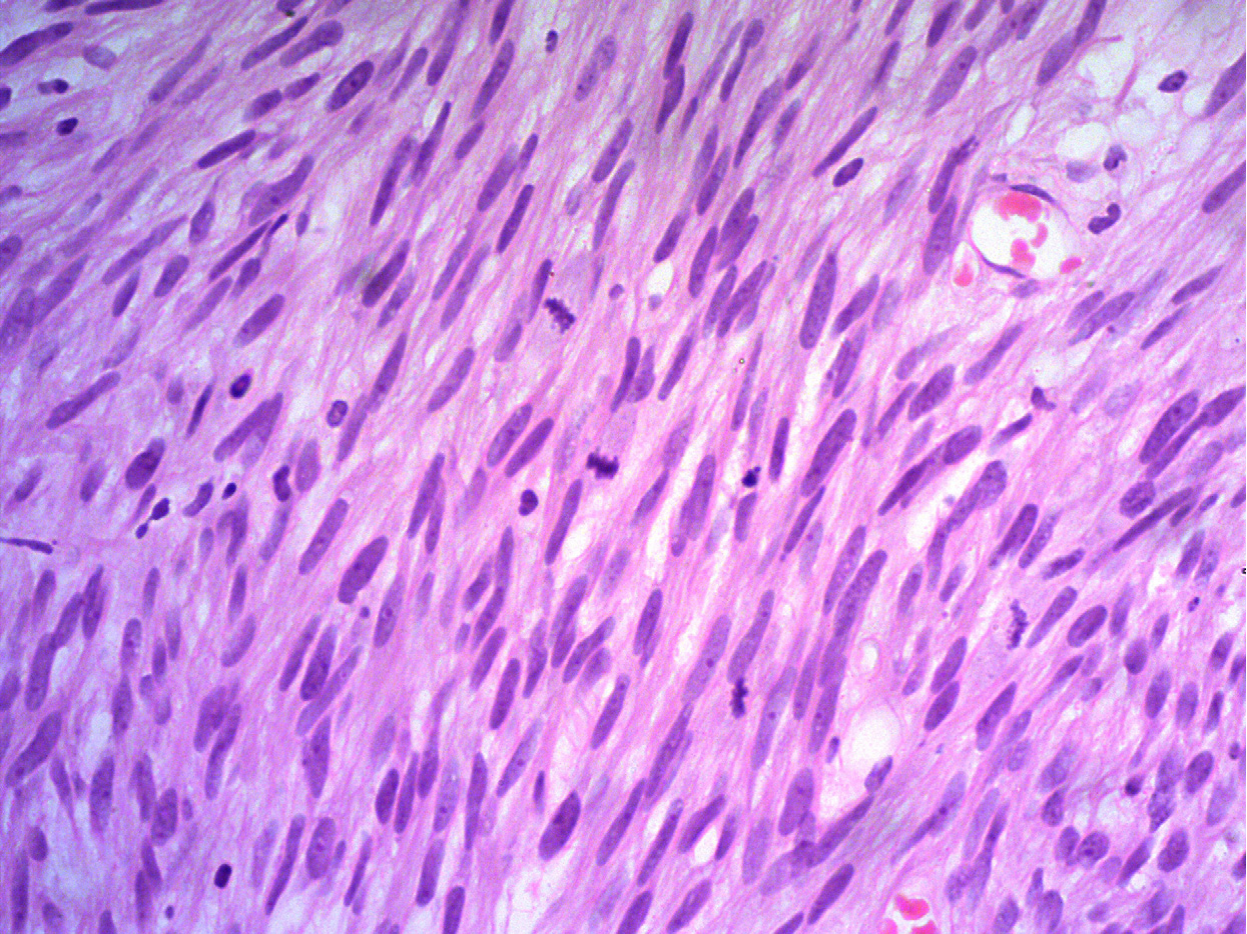
H and E staining viewed under × 40 showing a tumor composed of interlacing fascicles of spindle cells with elongated, blunt‐ended nuclei containing prominent nucleoli, and moderate abundant eosinophilic cytoplasm.

Cystoscopy showed a 2.5 cm solid exophytic bosselated bladder tumor with a smaller base on the right anterolateral wall of the bladder. A transurethral resection of bladder tumor (TURBT) was done. Histopathological analysis revealed polypoidal pieces of tissue resembling a spindle cell tumor, lined by normal urothelium (Fig. 2). The tumor was composed of interlacing fascicles of spindle cells with elongated, blunt‐ended nuclei containing prominent nucleoli and moderate abundant eosinophilic cytoplasm. Nuclear atypia was present focally with high mitotic activity (39/10 hpf). There was no evidence of tumor necrosis (Fig. 3). An immunohistochemical assay of desmin was carried out for further analysis. The tumor cells stained positively with smooth muscle actin and desmin and negatively with CD117. The Ki67 stain showed a proliferative index of about 70–80%. Therefore, a moderately differentiated leiomyosarcoma was diagnosed.

Based on the above findings, partial cystectomy and wide excision of the tumor were planned. A muscle dividing Pfannenstiel incision was made. A solid mass lesion was noted in the surface of the anterior bladder extending to the rectus muscle. The entire solid mass was excised after opening into the bladder with a large segment of the anterior wall of the bladder. Exploration of the peritoneal cavity did not reveal any pelvic lymph node involvement or evidence of metastasis. The recovery following surgery was uneventful.

She was followed up for 3.5 years with regular check cystoscopies and ultrasonography every 3 months in the first year and every 6 months thereafter, with no evidence of recurrence, and currently, she is well and asymptomatic.

## Discussion

The spectrum of bladder cancer is diverse, and the majority of cancers are urothelial in origin (90–95%) 3. Nonurothelial bladder cancers are known to occur in around 5–10% of all bladder cancers which are classified as epithelial and nonepithelial in origin 3.

Two retrospective reviews of mesenchymal genitourinary tumors revealed that sarcoma is the commonest mesenchymal malignancy of the bladder with leiomyosarcoma as the most common histological type 3, 4.

Of the total number of cases reported in the literature (*n* = 192), leiomyosarcomas were the commonest (50%) and 20% were rhabdomyosarcomas and others were carcinosarcomas, angiosarcomas, and osteosarcomas 1. No specific risk factors have been established; however, an association with retinoblastoma (RB) gene mutations, pelvic irradiation, and cyclophosphamide were reported in the literature 1, 5, 6, 7, 8, 9.

The review of the literature revealed that the overall prognosis is variable but generally poor 1, 2. In a recent series by Lee et al. 10, seven sarcomas of bladder were studied, and the estimated 5‐year survival rate was 73%. Rosser et al. 11 described 35 patients with high‐grade leiomyosarcoma treated exclusively with radical cystectomy with a 5‐year disease‐specific survival of 62%, and a recurrence rate of 34% at a median follow‐up of 38 months. A series by Lindberg et al. studied 34 cases of leiomyosarcomas of which only one tumor was well‐differentiated, while 17 were moderately differentiated and 16 were poorly differentiated. Of the 17 cases that were followed up for more than 12 months, an adverse outcome was seen in nine (53%) patients 12.

Because of the rarity of the disease, there is limited data on leiomyosarcomas and thus, there is no consensus on the management protocols. For patients with small lesions, minimally invasive procedures like transurethral resection or laser fulguration with or without chemoradiation have been utilized. Studies comparing a more radical approach compared to minimally invasive procedures are lacking 13.

In large tumors with an advanced stage at diagnosis, the treatment consists of radical cystectomy combined with chemoradiation. This procedure should include wide resection margins with at least a 2–3 cm depth free from tumor invasion. However, the procedure is associated with considerable morbidity and subsequent poor quality of life 14.

Partial cystectomy is currently being considered as a reliable option for management of various tumors including bladder sarcomas due to the functional preservation. A case report by Xu et al. 15 reported a 31‐year‐old woman with bladder leiomyosarcoma who was treated with partial cystectomy, and she had no recurrence over a 7‐year follow‐up period. The tumor was small (approximately 4 cm) and only confined to the muscle layer of the bladder wall and there was no local infiltration to the adjacent structures. Labanaris et al. 16 reported a young woman with a localized 1.2‐cm tumor without evidence of metastasis who was treated with partial cystectomy with adequate resection margins. The follow‐up after 8 months revealed no evidence of local recurrence or metastasis.

In the largest series to date of high‐grade bladder leiomyosarcoma (*n* = 35) managed by radical cystectomy and pelvic lymph node dissection at MD Anderson Cancer Center, only five patients (14.3%) were found to have microscopic disease in the regional lymph nodes at surgery 11. In view of the low positive yield following pelvic lymph node dissection, we waived the latter procedure from the surgical treatment, where the lymph nodes were palpably normal.

A close surveillance especially in the first year, with imaging and cystoscopy, is mandatory for early detection of local recurrence or metastasis 15. Although beneficial, adjuvant chemoradiotherapy is not essential immediately after partial cystectomy and may be reserved for those with recurrence 17. Local recurrences should be treated with systemic chemotherapy and/or pelvic external beam radiotherapy 18. Recent reports suggest that leiomyosarcoma of the bladder may have a better prognosis than once believed, with a 5‐year disease‐specific survival rate of >50%. Therefore further studies are needed to study the effectiveness of bladder preservation surgeries for treatment of selected cases as this involves preservation of bladder function and quality of life 15, 16.

## Conclusions

This case study described a locally advanced moderately differentiated leiomyosarcoma of the bladder without evidence of lymph node involvement of metastasis in CT scan, which was successfully treated with preservation of bladder function. Although bladder leiomyosarcoma has been regarded as an aggressive tumor with poor prognosis in the past, partial cystectomy and wide local excision may be considered a viable option for appropriate lesions without evidence of metastasis. This preserves the functional outcome and quality of life of an individual in comparison to radical cystectomy. However, in such cases, a good tumor‐free resection is extremely important, and meticulous follow‐up with imaging and endoscopy is mandatory to detect the recurrences early.

## Authorship

UJ, DMHF and KBH: contributed to collection of information and writing of the manuscript. MVCS and SASG: contributed to writing and final approval of the manuscript.

## Conflict of Interest

The authors declare that there is no conflict of interest.

## Consent

Informed written consent was obtained from the patient for publication.
